# Following Ribosome Footprints to Understand Translation at a Genome Wide Level

**DOI:** 10.1016/j.csbj.2018.04.001

**Published:** 2018-05-01

**Authors:** Guillermo Eastman, Pablo Smircich, José R. Sotelo-Silveira

**Affiliations:** a Department of Genomics, Instituto de Investigaciones Biológicas Clemente Estable, MEC, Av. Italia 3318, Montevideo, CP 11600, Uruguay; b Laboratory of Molecular Interactions, Facultad de Ciencias, Universidad de la República, Iguá 4225, Montevideo, CP 11400, Uruguay; c Department of Cell and Molecular Biology, Facultad de Ciencias, Universidad de la República, Iguá 4225, Montevideo, CP 11400, Uruguay

**Keywords:** Ribo-seq, Translation, Translatome, Transcriptome, Ribosome profiling

## Abstract

Protein translation is a key step in gene expression. The development of Ribosome Profiling has allowed the global analysis of this process at sub-codon resolution. In the last years the method has been applied to several models ranging from bacteria to mammalian cells yielding a surprising amount of insight on the mechanism and the regulation of translation. In this review we describe the key aspects of the experimental protocol and comment on the main conclusions raised in different models.

## 1 Introduction

The decreasing cost of obtaining Next Generation Sequencing (NGS) data [[1], [2], [3]] together with the huge information sets arising from these technologies is revolutionizing several research fields of life sciences (see an example in [4] or in disease biology [5,6]). Ingenuity is continuously leading to the development of new methods, a very interesting case is an application named Ribosome Profiling (RP), or Ribo-Seq, developed by Ingolia & Weissman in 2009 [7] where the deep sequencing of mRNA fragments covered by ribosomes during translation yielded an original view of translation at a genome wide scale. The footprints of active ribosomes are obtained using an RNAse protection assay, where controlled digestion generates small mRNA fragments/footprints of approximately 30 nucleotides [8]. Therefore, after data processing, translation can be observed at an unprecedented resolution in a variety of biological settings. Before performing the digestion, ribosomes are halted over the mRNAs using translation inhibitory drugs or by quick deep freezing the sample to avoid ribosome run-off. The resulting fragments, *i*.*e*. the ribosome footprints, are purified and used to construct sequencing libraries to feed short read sequencers. In this scenario, a transcriptome wide picture of the translating ribosomes location over mRNAs is obtained, together with an estimation of the mRNAs translation rates. These expression levels estimated by RP define what is called *translatome*, in analogy to the term transcriptome. Translatome estimations of gene expression levels correlate better with proteomic data than transcriptome-derived estimations (see below). This increased correlation evidences the existence of mechanisms operating in the control of translation that fine tune the synthesis of cellular proteins.

In the context of the rich data obtained in a RP experiment, an interesting outcome was the definition of two concepts: translational efficiency and periodicity. The first concept refers to how much an mRNA is translated considering the level of its coding mRNA, so it is an important parameter yielding information on translation regulation. Translational efficiency is calculated as the ratio between translation (derived from counts of footprints per mRNA) over transcription (derived from RNA-seq mRNA levels) of particular mRNA. The second, refers to the three bases mapping periodicity observed for the reads derived from footprints as a consequence of ribosome movement along mRNA. Since the ribosome moves codon by codon, the 5′-end of the ribosome footprints tend to map at the same position of each codon throughout the whole coding sequence.

Several aspects concerning protocol have been discussed, revised and modified since the original protocol was established. Some aim to adapt the protocol to different biological models, like eukaryotic or prokaryotic cells, specific tissues, *etc*. Other aspects have been intensely discussed, for example what the appropriate method to stop translation is or how to define the correct translation frame from ribosome footprints. Nevertheless, RP protocol is currently a widely used approach to study gene expression in different biological models from virus and bacteria to complex mammalian tissues (examples in [[9], [10], [11]]). In this mini-review we will discuss the main and critical steps in the RP protocol, its uses and main findings obtained in different biological models and the contributions to our knowledge of cellular and molecular biology.

## 2 Ribosome Profiling Protocol

### 2.1 Protocol Description

**Fig. 1:**
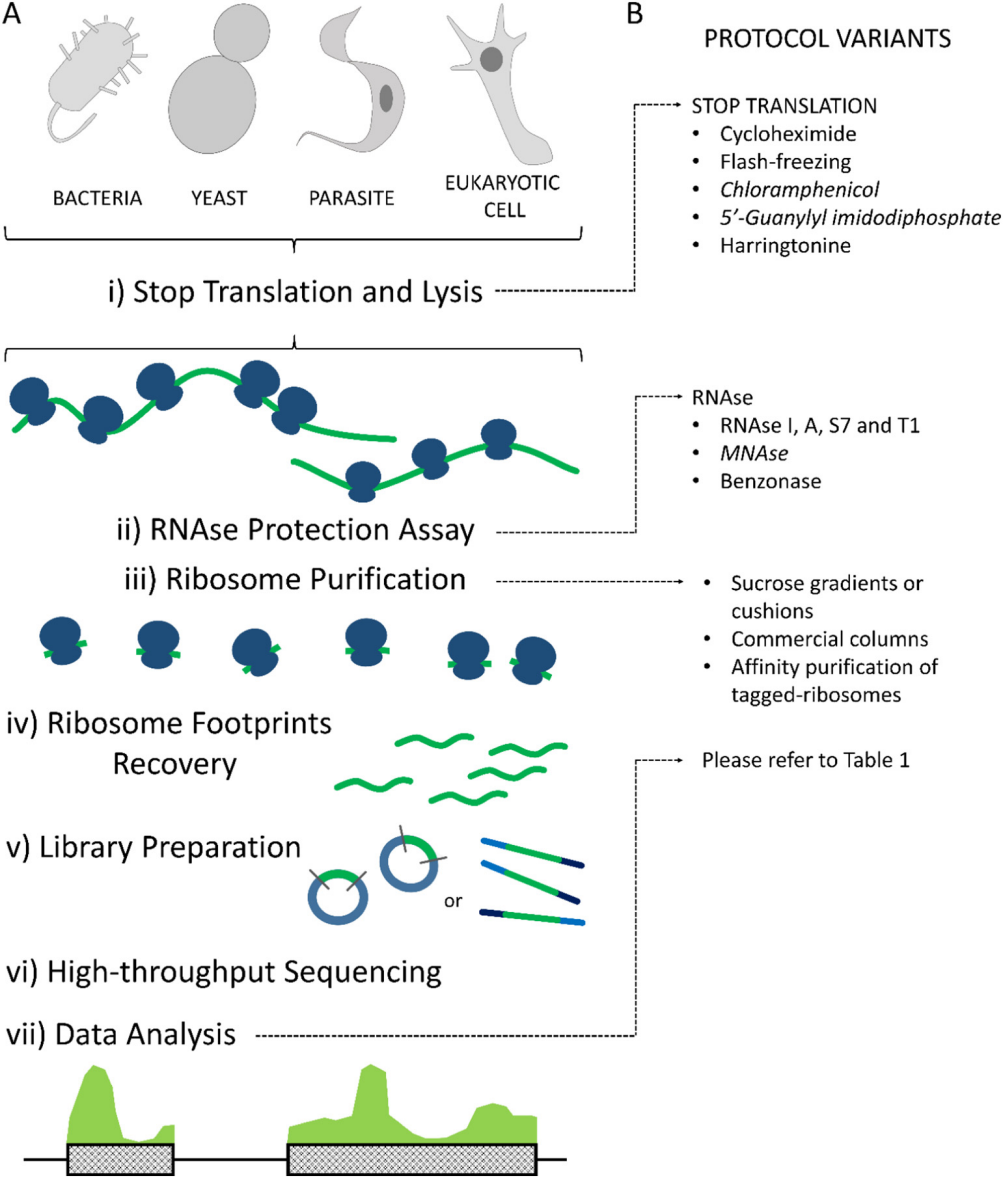
Ribosome Profiling protocol description. A general description of RP protocol is shown in A, representing the main steps described in the text. The protocol variants discussed are summarized in B, linked to the corresponding step where would be applied. Variants that correspond to prokaryotes are marked in italic.

Ribosome Profiling comprise mainly five steps: sample preparation, RNAse protection assay, isolation of ribosome footprints, high-throughput sequencing and bioinformatic analysis (Fig. 1A) [12]. Sample preparation refers to steps necessary to process the biological sample and obtain a post mitochondrial supernatant where lysis conditions ensure to preserve *in vivo* ribosome positioning and RNA integrity. Among others, alternative inputs could be tissue homogenates, isolated tagged ribosomes or a bacterial cell lysate. Critical aspects concerning this step are: ensuring enough biological material to produce quantifiable ribosome footprints and avoiding ribosome run-off. For the last, either drugs inhibitors of translation or physical methods like flash-freezing using liquid nitrogen and dry ice can be used. Indeed, fast freezing becomes crucial in cases where using translation inhibitors are to be avoided.

The RNAse protection assay, also called nuclease footprinting, is another critical step in RP protocol. Several RNAses had been used, mainly RNAse I and micrococcal nuclease (MNAse) in eukaryotic cell models and bacterial cells, respectively. At this step, controlling factors like reaction time and enzyme concentration are critical to ensure an appropriate mRNA digestion, for example it has been stablished that the ratio between RNA and RNAse controls footprints size [13].

The third step is one of the most laborious in terms of protocol. Different strategies had been used to isolate ribosome protected fragments or ribosome footprints, but all of them imply a ribosome/poly-ribosome purification step. Even though commercial columns are available to purify monosomes, the most used approach is the differential sedimentation of ribosomes through a sucrose cushion during ultracentrifugation. The use of this technique of subcellular fractionation ensures the purification of monosomes with bound ribosome footprints. Once monosomes are purified, a polyacrylamide gel electrophoresis in denaturing conditions is run to separate the complex sample by length. Using appropriate size markers, the gel is cut at the corresponding length of 28-30 nt using a dark field transilluminator, even if footprints are not visible as it is usually the case. After disrupting the gel slices, precipitation and re-purification of ribosome footprints, samples are ready to proceed to library preparation.

Library preparation implies a set of protocol steps common in many high-throughput sequencing experiments like end repair, 3′ adaptor ligation, reverse transcription and PAGE cDNA purification, circularization of cDNA and PCR amplification. After checking length and concentration of the ribosome footprints library, they can be submitted to sequencing according to user-preferred sequencing technologies. Due to footprints small size, neither long reads nor paired-end reads are needed. Nevertheless, due to ribosomal rRNA presence in the footprints fraction purified, depletion of rRNA, coupled with extra sequencing depth are usually needed.

Finally, the bioinformatic analysis of data is the most user-dependent step. A typical analysis would include quality control of raw reads, mapping, count normalization and gene expression levels estimation. It could also include, for example, differential gene expression analysis if two biological conditions are contrasted. Table 1 show a list of some of the software available to perform classical analysis over RP data. Nevertheless, how deeply the data is interrogated is on user's hands, here we will discuss some of these downstream analyses later.

**Table 1:** Software available to analyze, interpret and visualize RP-derived data. A list of some of the software used to analyze RP data is briefly described, indicating its main features and the adequate environment to use it.

Name	Functions/description	Enviroment	Ref.
riboSeqR	Parsing data, align reads, plotting functions, frameshift detection and inferring alternative ORFs.	R	[101]
RiboProfiling	Quality assessment, read start position recalibration, counting of reads on CDS, 3′UTR, and 5′UTR, plotting of count data: pairs, log fold-change, codon frequency and coverage assessment, principal component analysis on codon coverage.	R	[102]
RiboGalaxy	On-line tools for the analysis and visualization of ribo-seq data (some of them use riboSeqR)	Galaxy webserver	[103]
Plastid	A handful of scripts for common high-throughput sequencing and ribosome profiling analyses, like: determining P-sites offsets	Python Library	[104]
Ribomap	Generates isoform-level ribosome profiles from ribosome profiling data	Unix	[105]
RiboTraper	Identifies translated regions	Unix	[106]
Rfoot	Identifies RNA regions protected by non-ribosomal protein complex present in Ribo-Seq data	Perl	[107]
anota	Analysis of differential translation and results visualization	R	[108]
RiboDiff	An statistical tool to detect changes in protein translation efficiency	Unix	[109]
Xtail	An analysis pipeline that identifies differentially translated genes in pairwise comparisons	R	[110]
RiboTools	Detection of translational ambiguities, stop codon readthrough events and codon occupancy. Provides plots for the visualization of these events.	Galaxy webserver	[111]
Proteoformer	Genome-wide visualization of ribosome occupancy and a translation initiation site calling algorithm. A protein database can be incorporated to increase protein identification	Galaxy webserver	[112]
ORFscore	Small ORF identification	In SPECTtre [106]; python	[75]
ORF-RATER	Coding sequence annotation	Python	[113]
FLOSS	A metric for distinguishing between 80S footprints and nonribosomal sources using footprint size distributions	In SPECTtre [106]; python	[61]
tRanslatome	Analysis of transcriptome, translatome and proteome data: Differentially expressed genes detection, gene ontology enrichment comparison and analysis of regulatory elements	R	[114]
TranslatomeDB	Differential gene expression, translation ratio, elongation velocity index and translational efficiency. Also comparision with other RP experiments can be done	Online	[115]
systemPipeR	Filter/trim sequences, quality control, alignments, counting, peak detection, differentially expressed genes detection, enrichment, classification, several reports and graphs	R	[116]

### 2.2 Protocol Variants, User Decisions

Up to this point we have reviewed the main steps in RP protocol considering the classical approaches most used in literature. Henceforth we will mention some protocol variants and why they could be used if is necessary (Fig. 1B). Considering the chronological order of the protocol, we will start with one of the steps where more variants are described in the literature: how to stop translation at the moment the experimental design requires to do so. Efficient stop of translation avoids ribosome run off, sharpening the picture taken of the translatome at a given time point. In the original protocol, a classical translation inhibitor like cycloheximide was used to specifically target translation elongation. However, as it does not interfere with pre-initiation complex scanning and translation initiation, treatment with cycloheximide causes a significant accumulation of ribosomes at initiation sites of mRNAs actively translated. This could represent a source of bias since a lot of ribosome footprints will be generated by initiating ribosomes while elongation is stopped. This issue was highly covered in the literature, with some authors proposing that this accumulation is actually due to an enrichment of slow codons after the initiation and others are in line with the bias hypothesis that generates a skewed distribution. Alternatively, it is possible to stop translation using liquid nitrogen and dry ice [12]. In this scenario, ribosomes are flash-frozen and stopped just by reducing kinetic energy to a minimum. This alternative seems to not affect ribosome density and expression measurements but it's not the most extended approach, maybe because of availability of liquid nitrogen in the laboratories. When working with prokaryotes, besides flash-freezing, drugs like chloramphenicol and 5′-Guanylyl imidodiphosphate had been used [9]. Finally, it is worth mentioning that other drugs that target translation had been used to reveal specific aspects of translation. One of the most extended example is the use of harringtonine or the combined use of cycloheximide and harringtonine. Since harringtonine it is an inhibitor of translation initiation, the use of this drug alone could reveal translation initiation sites exclusively. Also, if harringtonine is first applied, and cycloheximide is applied after at different time points, it is possible to measure very specific translation properties like translation elongation speed [14,15].

The second step we mentioned it is the RNAse protection assay. In this step enzyme selection is critical [16]. In first place the biological model (eukaryotic or prokaryotic) already limits the options. In the literature, enzymes used for eukaryotic systems are mainly RNAse I, A, S7, T1 and MNAse, also used in prokaryotes. Since the method has been mainly applied to eukaryotic cells, RNAse I is the more common enzyme selected. In this case, the amount of RNA that is digested and other reaction conditions are well established, but when a *new* RNAse is being used, parameters like enzyme units and time of the digestion needs to be specifically determined to ensure a correct ribosome footprint production. It has been useful the use of enzymes, like Benzonase, or the above mentioned MNAse, that produce digestion products that allow a more straight forward ligation of the linkers required to prepare NGS molecular libraries [[17], [18], [19], [20]] simplifying the library preparation protocol.

Once cells are harvested, lysed and the RNAse protection assay is carried out, the next step is to collect ribosomes and specifically purify ribosome footprints. As we mentioned above, ribosome purification could be one of the most laborious step. Despite commercial columns are available to purify ribosomes, more classical strategies tend to be used, like monosome separation by ultracentrifugation in sucrose cushions or gradients. While sucrose gradients fractionation is challenging, sucrose cushions give similar results with less technical challenges. Other approaches to collect ribosomes are available, like genetic manipulation to add epitope tags to ribosomes, allowing affinity purification [[21], [22], [23]]. In any case, after ribosome isolation, footprints purification is the immediate follow step. Since the RNAses used are endonucleases, they digest “unprotected” mRNA while also cutting fragments of rRNA exposed in ribosome's surface. This digestion produces a very complex mix of RNA fragments of diverse length that is separated by a denaturing PAGE. Using appropriate size markers (26 and 34 nt), the band corresponding to ribosome footprints is excised from the gel and the RNA is isolated. Interestingly, a new population of small footprints of 20 nt in length was recently described [24]. This small population would not be recovered if we use the size markers mentioned above. In this context, depending on the experiment being performed and on the research goals, size selection can be modified accordingly.

Since the original sample contains a lot of ribosomes, a very important fraction of the generated fragments comes from rRNA. This contamination, still present in ribosome footprints expected band, is an important issue. One possible strategy is to continue with the protocol ignoring this contamination and go deep in sequencing to obtain enough mRNA derived sequences to achieve RNA-seq like coverage. However, this contamination can represent up to 90% of the sample, so a subtracting strategy is usually necessary. Ribosomal RNA removal can be achieved through streptavidin affinity purification using specific biotinylated rRNA probes available for mouse and human. If the biological model it is not mouse or human, synthesis of specific rRNA complementary oligos can be considered, provided by previous knowledge of the region of the rRNA protected in the model used. The later can be obtained by sequencing at low depth to determine the most abundant protected fragments derived from rRNA. Because different enzymes can produce different protected rRNA due to allosteric impediments or cleavage site sequence specificity, determining the identity of contaminating rRNAs could be necessary.

When footprints are collected, library construction and high-throughput sequencing are the next in line. Depending on the RNAse used, end repair could be necessary prior to linker ligation. While conventional protocols require PCR amplification and purification of the amplified PCR product by PAGE, as mentioned above some enzymes simplify these steps. Finally, sequencing is performed. While several platforms are available to perform high-throughput sequencing, long reads are not necessary as footprints are naturally short. Usually the depth of coverage to be achieved is dependent on how much rRNA is contaminating your footprints and how many mRNAs you will need to quantify.

Finally, data interpretation implies a complete *in silico* analysis (see Table 1), although this is the step more flexible and open to user aims, it represents several challenges due to the particular features of RP. For example, reads are short in length, may have relatively high error rates and depending on library construction protocol could have high bias. Also, some fragments tend to be enriched, because accumulated ribosomes at translation initiation sites or pausing sites, leading to high read counts. Beyond this, most of the available tool to process and analyze experiments of RNA-Seq are suitable to use analyzing data from RP, specifically the ones used to short length reads and/or single-end reads. Nevertheless, some aspects need to be considered due to the peculiarities of the data set analyzed. For example, gene isoforms studies are difficult since ribosome footprints are short reads and mapping over splice junctions tend to be unreliable. Briefly, bioinformatic analysis implies in general: quality and adaptor trimming, mapping against a specific data base of rRNA or ncRNAs to remove contamination, unmapped reads are aligned to an mRNA data base, counting reads, normalize counts and proceed to check statistical differences between conditions. As said above, diverse analysis can be done with data, just to mention some: check footprints periodicity, upstream Open Reading Frame (uORF) search, detection of different translation initiation sites, codon usage and search for translation pauses, among others. Even though general-purpose RNA-seq tools may be suitable, some specific software has been developed to apply to RP data set that explicitly consider the influence of transcript levels on translatome determinations (see examples in Table 1).

## 3 Biological Models and Contributions

Up to date, the RP protocol has been applied to a large variety of biological models from viruses and bacteria to yeast, mammalian cells and tissues, and embryos. In this section we will present the main contributions done in each model, and also what we have learned about the translation mechanism using this methodology. In addition, in Table 2 several RP works were grouped by the main topic analyzed, indicating in each case the different organisms used.

**Table 2:** Brief summary of RP works in several models, grouped by the main analyzed topic.

Topic	Organism	Ref.
Genomic/translation characterization	Virus	[11,[86], [87], [88]]
	*Mycobacterium abscessus*	[35]
	Mammalian cells	[14]
Translation initiation sites	*Caulobacter crescentus*	[26]
	Mammalian cells	[59]
Translation elongation	*Saccharomyces cerevisiae*	[24]
	*Caenorhabditis elegan*	[79]
Translational pausing	*Escherichia coli*	[9,27,32]
	*Bacillus subtilis*	[9]
	*Saccharomyces cerevisiae*	[46,67]
Codon usage	*Escherichia coli*	[37]
	*Saccharomyces cerevisiae*	[47,49,50]
Small ORF	*Saccharomyces cerevisiae*	[51]
	Zebra fish	[75]
	*Drosophila melanogaster*	[77]
	Mammalian cells	[65]
Translation dynamics on different stages	*Plasmodium falciparum*	[81,82]
	*Trypanosoma cruzi*	[17]
	*Trypanosoma brucei*	[83,84]
Stress response	*Escherichia coli*	[41]
	*Mycoplasma gallisepticum*	[34]
	*Arabidopsis thaliana*	[80]
	*Saccharomyces cerevisiae*	[7]
lncRNAs translation	Mammalian cells	[[60], [61], [62], [63], [64]]

### 3.1 Bacteria: Translational Pausing, Codon Use and Antibiotics

In bacteria, ribosome profiling was applied in first place to *Escherichia coli* and *Bacillus subtilis* [9] to study the causes of translational pausing. The authors observed that the presence of Shine-Dalgarno-like features in coding sequences are the major determinants of translation rates in these models. Instead of codon usage or the presence of rare tRNAs, interactions between rRNA and these Shine-Dalgarno-like features in mRNA can impact on ribosomal movement along mRNA, which in turn affect footprints location and abundance [25]. Later, Schrader et al. [26] also applied RP, in *Caulobacter crescentus* and arrived to the same conclusion: ribosomes tend to pause at internal Shine-Dalgarno-like sequences in coding genes. Although the later hypothesis regarding underling mechanisms of translation pausing in bacteria is still controversial (see an example in [27]), with authors supporting classical hypothesis of tRNA abundance as main modulator of translation speed, this is still a new possible mechanism for regulating translation uncovered by the RP strategy.

In another study Oh et al. [28], investigated a chaperone trigger factor and how this protein regulates outer membrane proteins, using a RP protocol modified later in [29]. Balakrishnan et al. [30] studied translation initiation on *E*. *coli* using RP, while translation elongation was covered by Elgamal et al. [31], where authors find translational pauses associated to elongation factor P and amino acids motifs upstream to ribosome P-site (also found in [32]). Other bacteria where RP was applied are *Mycoplasma gallisepticum* [33,34], *Mycobacterium abscessu* [35] and *Staphylococcus aureus* [36]. RP as a powerful technique to measure translation rates at subcodon resolution, has allowed scientist to focus on the relationship between translation efficiency and codon usage deriving in the optimization bacterial vectors for expression of heterologous recombinant proteins [37,38].

Also, RP has given new insights on the antibiotics mechanisms to inhibit translation [39]. Other studies have been using RP to investigate mechanisms for biofilm formation in *B*. *subtilis* [40], ethanol effects on translation [41] and mRNA cleavage by the endonuclease RelE [42].

### 3.2 Yeast: Start Codons, uORFs and Translational Pauses

Since RP was firstly described in the budding yeast *Saccharomyces cerevisiae* [7], a lot of research has been done using this model and by re-analyzing that public data sets generated. In the original article, Ingolia et al. [7] explored translation response to starvation. In this seminal paper the terms translation efficiency and periodicity were defined for first time in this context (see Introduction). While translation efficiency is usually calculated in every experiment using RP, periodicity is not assessed so often, because it depends on RNAse amount used and digestion time.

**Fig. 2:**
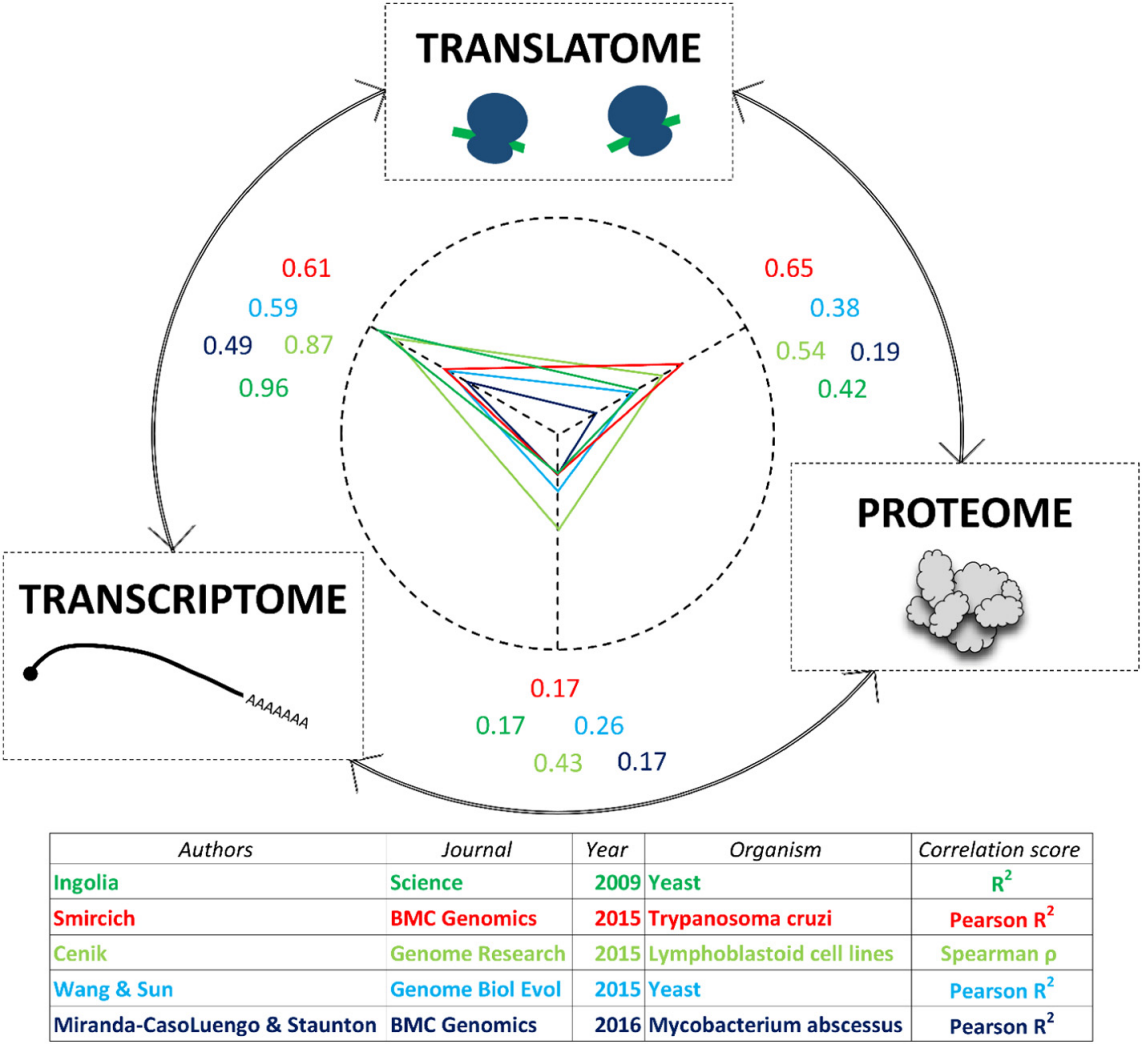
Correlations among RNA-Seq, RP and proteome-derived expression data sets. Genome-wide correlations of individual gene expression levels estimated by RNA-Seq, RP and proteome techniques are shown. Each correlation value is referenced to its corresponding author, indicating also journal, year, organism involved and correlation test used, by the same color code.

For first time, integrating all data obtained, correlations between expression levels estimated by RNA-Seq (transcriptional levels), RP (translational levels) and proteomics (protein levels) could be obtained, reflecting the contribution of translational regulation in the fine tuning of final proteins levels (please see examples in Fig. 2). In this sense, other efforts have been made to correlate translation ratios and protein abundance. For example, Wang et al. [43] by incorporating mRNA length as a key factor, found a strong multivariate linear correlation between protein levels and translation ratios estimated by ribosome-nascent chain complex sequencing (RNC-Seq). The correlation between translational and protein levels estimated by RP and proteomics, respectively, may be improved if elongation velocity index are incorporated in the analysis, according to the authors [44] (please see Section 3.3.2).

Also, start codons were also precisely determined in this work, and initiation at non-AUG codons was observed as response to starvation. In the same way, detection of ribosome footprints at 5’-UTRs reveals translational activity in these regions mainly explained by the presence of uORFs. In this way, a new approach to uORF study and its relationship with translation regulation was stablished, revealing a completely new and complex field previously not covered in detail.

To highlight some of these contributions yeast models provided, we can mention that distinct population of ribosome footprints were discovered and were assigned to distinct stages of translating ribosomes [24]. Furthermore, 80S ribosomes (monosomes) were detected as translationally active, translating specific mRNAs encoding low abundance and regulatory proteins, among others [45]. In addition, codon usage, tRNA levels and how they influence translation was highly covered [[46], [47], [48], [49], [50]]. The hypothesis that arise more strongly in yeast is that biochemical interactions between the nascent peptide and the ribosomal exit tunnel (in particular the initial part of the tunnel) are major determinants on ribosome stalling [46]. A stalling signal of proline and arginine was detected, as others showed for bacteria [31,32]. On the other hand, also the correlation between tRNA concentrations and codon decoding time was evaluated, finding a significant negative correlation, supporting the idea that translation efficiency is influenced by tRNAs levels in the cells [48]. Also, RP was used to explore the genome-wide translation of small ORFs (<100 amino acids) and long non coding RNAs (lncRNAs) [51], ribosome rescue in 3′-UTR [52], the yeast meiotic program with important contributions to the area [53], and also how translation contributes to regulate gene-expression in yeast in an evolutionary view [54].

### 3.3 Mammalian Cells: uORFs, Pauses, Initiation Sites and lncRNAs

In mammalian cells, the first study carried out applied RP strategy to reveal aspects of microRNA's (miRNA) function in the cell [55]. The authors observed that miRNA predominantly affect mRNA levels, with only a modest influence on translational efficiency. This study revealed for first time that mRNA destabilization is the major consequence of miRNA regulation. So, from here to the end of this section we will present some interesting research and their results in mammalian cells mainly, but also in other eukaryotic models.

A significant study in terms of results, conclusions and repercussions, was done by the group who publish the RP protocol, but using mouse embryonic stem cells (mESC) [14]. In this model, the authors identified thousands of pause sites and unannotated translation products like amino-terminal extension and uORFs with potential regulatory roles. In parallel, authors combine harringtonine and cycloheximide use to monitoring kinetic of translation as we describe below, evidencing a ribosome translation rate of 5.6 amino acids per second, consistent with previous values [56], and that is independent of length, protein abundance, classes of mRNAs or codon use.

#### 3.3.1 uORFs

Ingolia et al. [14], using harringtonine in mESC, could identify translation initiation sites, where AUG was present in almost 75% of canonical sites, but in <25% in upstream sites, where others near-AUG codons were observed, like CUG and GUG (see also Section 3.3.3). Considering the initiation site defined, the reading frame associated was also investigated and classified based on their relationship to the annotated ORF. In this characterization, many uORF were detected, as well as alternate protein products with amino-terminal extensions or truncations. The authors also study the widespread translation of uORFs detected and their change during differentiation, highlighting the important regulatory role that these elements have affecting translation, particularly when the cell is under stress conditions [57]. A well-known example is the uORF translation regulation that affects GCN4 expression in yeast under starvation [7].

#### 3.3.2 Translational Pauses and Elongation Speed

Regarding translational stall sites, Ingolia et al. [14] observed in mESC a consensus peptide motif of glutamate (preferentially GAA codon) or aspartate in the A site of pauses, preceded by a proline or glycine, and then another proline (preferentially represented by CC[A/T] codons); while no evidence of rare codons enrichment was seen in pausing sites. Also, Dana and Tuller [58], re-analyzed the data focusing on elongation speed and ribosome profiles. Their analysis suggest that elongation speed is indeed determined by the tRNA pool, local mRNA folding and local charge of amino acids encoded; an idea that seems to be extended in different articles, as was mentioned before (see also [46]). Nevertheless, the authors mention that they detect an unknown source of biases in the data that can interfere in ribosome profiles over mRNAs. Nevertheless, by experimentally assessing elongation velocity, recently Lian & Guo et al. [44] found that these general conclusions we have described may not be applicable to all individual cases. In this work, information from RNA-Seq, RP and also RNC-mRNA was used to define and calculate an elongation velocity index at individual genes in human cells. This index was correlated with several mRNA features and also with biological conditions, where authors find an elongation speed deceleration on malignant phenotype associated genes.

#### 3.3.3 Translation Initiation Sites (TIS)

Combining more data sets, Michel et al. [59] designed a method to estimate the probability of ribosomes initiating at individual start codons. This tool is able to discriminate between weak or strong initiation sites based on the accepted leaky scanning model of translation initiation in eukaryotes. For example, analyzing the codon preference in TIS in human and mouse, a > 50% of AUG TIS and also almost 50% of AUG preference in downstream TIS was observed. Composition of upstream TIS was more diverse: 25% are AUG codons, 30% CUG and 40% include other AUG-variants like UUG, GUG, AGG, ACG, among others [15].

#### 3.3.4 Long Non Coding RNAs

With no doubt, another striking finding of the work done by Ingolia et al. [14] in mESC was the detection of high levels of ribosome footprints in long intragenic noncoding RNAs (lincRNAs), with marked initiation sites evidenced by harringtonine. They classify these RNAs as sprcRNA: short, polycistronic ribosome-associated coding RNAs. If lincRNA encode or not a message to be translated by the ribosomes is a matter addressed specifically in two publications [60,61]. Guttman et al. [60] defined a Ribosome Release Score, that discriminate between coding and noncoding transcripts. Using this score, authors claim that the ribosome occupancy observed on lincRNAs *per se* is not an indicator of active translation and describe possible reasons why noncoding RNAs show ribosome footprints. One of these possibilities is that these footprints actually come from ribonucleo protein particles or others RNA-protein complexes. Alternatively, footprints could be generated by real engagement of ribosomes over ncRNAs that will not be functional at the end. This interesting controversy was going to take an unexpected turn when just over a year later, again Ingolia and Weissman described a different metric to analyze footprints, that now classify lncRNAs as coding [61]. This new metric called FLOSS (fragment length organization similarity score) measure the magnitude of disagreement between length distribution of a set of transcripts of interest and annotated protein-coding transcripts. Based on FLOSS and other lines of evidence, the authors proposed that lncRNAs has ribosome footprints that show features of translation. In addition alternative hypothesis were discussed: *i*) translating ribosome could act as a potent helicase to remodel RNA structures and remove RNA-binding proteins; *ii*) translated sequences may also act as cis-acting elements over lncRNAs that originate them and *iii*) the authors discuss about a possible contribution of the proteins synthetized by noncanonical translation to serve as possible antigens presented to the cellular immune system, expanding the universe of epitopes either in a viral infection or in a tumoral context. In any case, the fact that some ncRNAs are associated with ribosomes, translationally active or not, generates both challenging and interesting questions that wait to be answered (see examples in [[62], [63], [64]]).

Using the data produced by RP on mESC, a lot of downstream analysis has been conducted. For example, an approach to search and predict putatively functional small ORF was developed to identify new classes of bioactive peptides [65]. Another example is the work done by Zupanic et al. [66], where the authors developed a method to study mRNA translation regulation analyzing individual ribosome profiles. Incorporating RNA-Seq data to correct bias and artifacts, they look for changes in ribosome density along mRNAs to detect mechanisms of regulation, like premature termination or new transcript isoforms.

Regarding bias, several articles have studied this important issue on RP data. Some improvements have been done in terms of understand the bias source, and be able to correct it accurately [58,67,68].

The movement of the ribosome over the mRNA has been studied analyzing in deep mapping periodicity leading to undercover mechanisms underlying translational frameshifts [59]. Also regions in the human genome that are dually decoded were identified (~1% of human genome approx.), either from different mRNAs as from the same, expanding our vision about translation regulation and even about central dogma [4,59].

In HeLa cells, RP was applied to explore the translational landscape of cell cycle, and a widespread translation regulation was seen over cell cycle progression [69,70]. Surprisingly, evidence of functional bicistronic mRNAs with antiviral functions in the innate immune system was also revealed by RP in a human cell line [71]. Furthermore RP was used in humans to investigate genetic variants in lymphoblastoid cells derived from a diverse group of 30 individuals and how some genetic differences may modulate ribosome occupancy [72].

The mTOR pathway is a very important target of different drugs and has been implicated in several diseases, including cancer. Since this complex regulates cell growth and proliferation by regulating mRNA translation, it is interesting to use RP protocol to elucidate translation control executed by mTOR. This was done by Sabatini's [73] and Ruggero's [74] labs, and what they found was a surprising simple model of the mRNA features that mediates mTORC1-dependent translation: an established 5′ terminal oligopyrimidine (TOP) motifs. 5′-UTR length or complexity was not associated with mTORC1 translation regulation. The later also identify another motif called PRTE (pyrimidine-rich translational element) in 5′-UTR of mTOR targets mRNAs, which in conjunction with TOP motif were founded in almost 90% of mTOR-sensitive genes. A common result of both works, which undoubtedly draws attention, is the low regulatory spectrum found in terms of number of messengers: mTOR-regulated mRNAs were 253 and 144, respectively for each publication, a low number of targets considering the central role of mTOR pathway in cellular metabolism and previous results of translation control resolution using RP. It is still an open question whether this number changes in different cell types or conditions, since there are still several factors downstream of mTOR that influences what is being translated.

### 3.4 Others Biological Models: Zebrafish, Drosophila, *C*. *elegans*, Trypanosomatids and Virus

Besides bacteria, yeast and mammalian cell lines, the RP method was used to study translation regulation in others biological models as zebrafish [63,75], the fruit fly *Drosophila* [[76], [77], [78]], *C*. *elegans* [79], *Arabidopsis* [80] and also parasites like *Plasmodium falciparum* [81,82], *Trypanosoma brucei* [83,84] and *T*. *cruzi* [17]. Trypanosomatids undergo a complex life cycle with several distinct developmental forms, each having particular morphologic and metabolic profiles. However, these organisms accomplish the associated gene expression changes without transcriptional control [85]. Indeed, translation regulation proved to be a key mechanism controlling protein levels as revealed by drastic changes in translational efficiency for many developmentally regulated genes. For instance, the transition from a dividing to a non-diving parasite form was accompanied by a decrease in the translational efficiency of ribosomal proteins which in turn may explain the observed global decrease in protein synthesis. However, proteins required in the non-dividing stage scape this general trend and are actively translated as shown for the trans-sialidase family of virulence factors in *T*. *cruzi* [17]. Besides, the data allowed the curation of the available genomes in these non-model organisms [84].

Also, RP was applied to study translation in virus like human cytomegalovirus and Kaposi's sarcoma-associated herpesvirus, both herpesvirus, and also in Cricket paralysis virus and Influenza A virus (see [11,[86], [87], [88]], respectively).

## 4 Applications, Challenges and Perspectives

Besides classical applications we have been discussing above, like determine translation gene expression levels, pause associated motifs, codon translational rates, uORF and frameshift events detection, among others, here we will mention specific protocols that had evolved from initial RP experiments, like how to determine TIS by Qian lab [15,89]. In first place, they describe an approach named global translation initiation sequencing (or GTI-Seq) that combine the use of lactimidomycin and cycloheximide to detect both initiation and elongation ribosomes along transcripts, in human and mouse. The other, but similar approach, named QTI-Seq (Quantitative Translation Initiation Sequencing) evaluating not only TIS qualitatively, but also quantitatively, so statistical comparisons can be made between two conditions. In bacteria also exist an approach to identify TIS genome-wide named tetracycline-inhibited RP [90].

Research on mitochondrial and chloroplast translation is also possible using RP [[91], [92], [93]]. Recently, an specific application of RP named mitochondrial ribosome (mitoribosome) profiling was developed [94]. In this case, the approach developed in yeast consist in the immunoprecipitation of mitoribosomes from cell lysates to perform RNAse digestion. A similar approach but targeting reticulum-bound ribosomes was also used, in mammalian cells, to study translation related to intracellular traffic of membranes [19].

Throughout this minireview we have shown how the RP method has provided the scientific community with a powerful system to study the translation mechanisms and regulation, and more generally a more complete picture of regulation of gene expression in several models.

However even when the seminal paper will turn 10 years old next year many aspects of the technique are not completely resolved, as can be shown in the continuous development of new experimental protocols and analysis tools. Variations of the method are emerging to address particular cases, such as the development of protocols to assess localized translation. For instance, Williams et al. [95] reported proximity-specific ribosome profiling to target translation of nuclear encoded mitochondrial genes by tagging ribosomes in close contact with the outer mitochondrial membrane. In this context, localized translation can be investigated in more difficult scenarios like protein synthesis in neuronal projections [[96], [97], [98]], specifically Holt lab performed ribosome tagging and analyzed mRNA associated to tagged polysomes in the pre-synaptic area of a minute portion of the brain [99] but the low input in mRNA would impair ribosome profiling. So, it is still necessary to develop methods that will allow the study of systems where input material is a limiting factor. Some work is starting to appear in this field [100].

Some intriguing questions have not been yet pursued, particularly there are just a few reports where RP has been applied to disturbed systems, for example drug treated cells or pathological cells, as neurodegenerative diseases tissue or cancer cells where translation me be playing a key role in the etiology of the abnormal molecular processes.

## References

[1] Sboner A., Mu X.J., Greenbaum D., Auerbach R.K., Gerstein M.B. (2011). The real cost of sequencing: higher than you think!. Genome Biol.

[2] Buermans H.P.J., Dunnen den J.T. (2014). Next generation sequencing technology: advances and applications. Biochim Biophys Acta.

[3] Muir P., Li S., Lou S., Wang D., Spakowicz D.J., Salichos L. (2016). Erratum to: the real cost of sequencing: scaling computation to keep pace with data generation. Genome Biol.

[4] Mouilleron H., Delcourt V., Roucou X. (2016). Death of a dogma: eukaryotic mRNAs can code for more than one protein. Nucleic Acids Res.

[5] Vogelstein B., Papadopoulos N., Velculescu V.E., Zhou S., Diaz L.A., Kinzler K.W. (2013). Cancer genome landscapes. Science.

[6] Campbell B.B., Light N., Fabrizio D., Zatzman M., Fuligni F., de Borja R. (2017). Comprehensive analysis of hypermutation in human cancer. Cell.

[7] Ingolia N.T., Ghaemmaghami S., Newman J.R.S., Weissman J.S. (2009). Genome-wide analysis in vivo of translation with nucleotide resolution using ribosome profiling. Science.

[8] Steitz J.A. (1969). Polypeptide chain initiation: nucleotide sequences of the three ribosomal binding sites in bacteriophage R17 RNA. Nature.

[9] Li G.-W., Oh E., Weissman J.S. (2012). The anti-Shine-Dalgarno sequence drives translational pausing and codon choice in bacteria. Nature.

[10] Castelo-Szekely V., Arpat A.B., Janich P., Gatfield D. (2017). Translational contributions to tissue specificity in rhythmic and constitutive gene expression. Genome Biol.

[11] Stern-Ginossar N., Weisburd B., Michalski A., Le V.T.K., Hein M.Y., Huang S.-X. (2012). Decoding human cytomegalovirus. Science.

[12] Ingolia N.T., Brar G.A., Rouskin S., McGeachy A.M., Weissman J.S. (2012). The ribosome profiling strategy for monitoring translation in vivo by deep sequencing of ribosome-protected mRNA fragments. Nat Protoc.

[13] McGlincy N.J., Ingolia N.T. (2017). Transcriptome-wide measurement of translation by ribosome profiling. Methods.

[14] Ingolia N.T., Lareau L.F., Weissman J.S. (2011). Ribosome profiling of mouse embryonic stem cells reveals the complexity and dynamics of mammalian proteomes. Cell.

[15] Lee S., Liu B., Lee S., Huang S.-X., Shen B., Qian S.-B. (2012). Global mapping of translation initiation sites in mammalian cells at single-nucleotide resolution. Proc Natl Acad Sci U S A.

[16] Gerashchenko M.V., Gladyshev V.N. (2017). Ribonuclease selection for ribosome profiling. Nucleic Acids Res.

[17] Smircich P., Eastman G., Bispo S., Duhagon M.A., Guerra-Slompo E.P., Garat B. (2015). Ribosome profiling reveals translation control as a key mechanism generating differential gene expression in Trypanosoma cruzi. BMC Genomics.

[18] Marcon B.H., Holetz F.B., Eastman G., Origa-Alves A.C., Amorós M., de Aguiar A.M. (2017). Downregulation of the protein synthesis machinery is a major regulatory event during early adipogenic differentiation of human adipose-derived stromal cells. Stem Cell Res.

[19] Reid D.W., Nicchitta C.V. (2012). Primary role for endoplasmic reticulum-bound ribosomes in cellular translation identified by ribosome profiling. J Biol Chem.

[20] Reid D.W., Shenolikar S., Nicchitta C.V. (2015). Simple and inexpensive ribosome profiling analysis of mRNA translation. Methods.

[21] Sanz E., Yang L., Su T., Morris D.R., McKnight G.S., Amieux P.S. (2009). Cell-type-specific isolation of ribosome-associated mRNA from complex tissues. Proc Natl Acad Sci U S A.

[22] Heiman M., Schaefer A., Gong S., Peterson J.D., Day M., Ramsey K.E. (2008). A translational profiling approach for the molecular characterization of CNS cell types. Cell.

[23] Shigeoka T., Jung J., Holt C.E., Jung H. (1649). Axon-TRAP-RiboTag: affinity purification of translated mRNAs from neuronal axons in mouse in vivo. Methods Mol Biol.

[24] Lareau L.F., Hite D.H., Hogan G.J., Brown P.O. (2014). Distinct stages of the translation elongation cycle revealed by sequencing ribosome-protected mRNA fragments. Elife.

[25] O'Connor P.B.F., Li G.-W., Weissman J.S., Atkins J.F., Baranov P.V. (2013). rRNA:mRNA pairing alters the length and the symmetry of mRNA-protected fragments in ribosome profiling experiments. Bioinformatics.

[26] Schrader J.M., Zhou B., Li G.-W., Lasker K., Childers W.S., Williams B. (2014). The coding and noncoding architecture of the Caulobacter crescentus genome. PLoS Genet.

[27] Mohammad F., Woolstenhulme C.J., Green R., Buskirk A.R. (2016). Clarifying the translational pausing landscape in bacteria by ribosome profiling. Cell Rep.

[28] Oh E., Becker A.H., Sandikci A., Huber D., Chaba R., Gloge F. (2011). Selective ribosome profiling reveals the cotranslational chaperone action of trigger factor in vivo. Cell.

[29] Latif H., Szubin R., Tan J., Brunk E., Lechner A., Zengler K. (2015). A streamlined ribosome profiling protocol for the characterization of microorganisms. Biotechniques.

[30] Balakrishnan R., Oman K., Shoji S., Bundschuh R., Fredrick K. (2014). The conserved GTPase LepA contributes mainly to translation initiation in Escherichia coli. Nucleic Acids Res.

[31] Elgamal S., Katz A., Hersch S.J., Newsom D., White P., Navarre W.W. (2014). EF-P dependent pauses integrate proximal and distal signals during translation. PLoS Genet.

[32] Woolstenhulme C.J., Guydosh N.R., Green R., Buskirk A.R. (2015). High-precision analysis of translational pausing by ribosome profiling in bacteria lacking EFP. Cell Rep.

[33] Fisunov G.Y., Evsyutina D.V., Arzamasov A.A., Butenko I.O., Govorun V.M. (2015). Profiling of mycoplasma gallisepticum ribosomes. Acta Nat.

[34] Fisunov G.Y., Evsyutina D.V., Garanina I.A., Arzamasov A.A., Butenko I.O., Altukhov I.A. (2017). Ribosome profiling reveals an adaptation strategy of reduced bacterium to acute stress. Biochimie.

[35] Miranda-CasoLuengo A.A., Staunton P.M., Dinan A.M., Lohan A.J., Loftus B.J. (2016). Functional characterization of the Mycobacterium abscessus genome coupled with condition specific transcriptomics reveals conserved molecular strategies for host adaptation and persistence. BMC Genomics.

[36] Basu A., M-NF Yap (2016). Ribosome hibernation factor promotes staphylococcal survival and differentially represses translation. Nucleic Acids Res.

[37] Nakahigashi K., Takai Y., Shiwa Y., Wada M., Honma M., Yoshikawa H. (2014). Effect of codon adaptation on codon-level and gene-level translation efficiency in vivo. BMC Genomics.

[38] Li G.-W. (2015). How do bacteria tune translation efficiency?. Curr Opin Microbiol.

[39] Marks J., Kannan K., Roncase E.J., Klepacki D., Kefi A., Orelle C. (2016). Context-specific inhibition of translation by ribosomal antibiotics targeting the peptidyl transferase center. Proc Natl Acad Sci U S A.

[40] Subramaniam A.R., Deloughery A., Bradshaw N., Chen Y., O'Shea E., Losick R. (2013). A serine sensor for multicellularity in a bacterium. Elife.

[41] Haft R.J.F., Keating D.H., Schwaegler T., Schwalbach M.S., Vinokur J., Tremaine M. (2014). Correcting direct effects of ethanol on translation and transcription machinery confers ethanol tolerance in bacteria. Proc Natl Acad Sci U S A.

[42] Hwang J.-Y., Buskirk A.R. (2017). A ribosome profiling study of mRNA cleavage by the endonuclease RelE. Nucleic Acids Res.

[43] Wang T., Cui Y., Jin J., Guo J., Wang G., Yin X. (2013). Translating mRNAs strongly correlate to proteins in a multivariate manner and their translation ratios are phenotype specific. Nucleic Acids Res.

[44] Lian X., Guo J., Gu W., Cui Y., Zhong J., Jin J. (2016). Genome-wide and experimental resolution of relative translation elongation speed at individual gene level in human cells. PLoS Genet.

[45] Heyer E.E., Moore M.J. (2016). Redefining the translational status of 80S monosomes. Cell.

[46] Sabi R., Tuller T. (2015). A comparative genomics study on the effect of individual amino acids on ribosome stalling. BMC Genomics.

[47] Pop C., Rouskin S., Ingolia N.T., Han L., Phizicky E.M., Weissman J.S. (2014). Causal signals between codon bias, mRNA structure, and the efficiency of translation and elongation. Mol Syst Biol.

[48] Dana A., Tuller T. (2014). The effect of tRNA levels on decoding times of mRNA codons. Nucleic Acids Res.

[49] Gardin J., Yeasmin R., Yurovsky A., Cai Y., Skiena S., Futcher B. (2014). Measurement of average decoding rates of the 61 sense codons in vivo. Elife.

[50] Gorochowski T.E., Ignatova Z., Bovenberg R.A.L., Roubos J.A. (2015). Trade-offs between tRNA abundance and mRNA secondary structure support smoothing of translation elongation rate. Nucleic Acids Res.

[51] Smith J.E., Alvarez-Dominguez J.R., Kline N., Huynh N.J., Geisler S., Hu W. (2014). Translation of small open reading frames within unannotated RNA transcripts in Saccharomyces cerevisiae. Cell Rep.

[52] Guydosh N.R., Green R. (2014). Dom34 rescues ribosomes in 3′ untranslated regions. Cell.

[53] Brar G.A., Yassour M., Friedman N., Regev A., Ingolia N.T., Weissman J.S. (2012). High-resolution view of the yeast meiotic program revealed by ribosome profiling. Science.

[54] McManus C.J., May G.E., Spealman P., Shteyman A. (2014). Ribosome profiling reveals post-transcriptional buffering of divergent gene expression in yeast. Genome Res.

[55] Guo H., Ingolia N.T., Weissman J.S., Bartel D.P. (2010). Mammalian microRNAs predominantly act to decrease target mRNA levels. Nature.

[56] Boström K., Wettesten M., Borén J., Bondjers G., Wiklund O., Olofsson S.O. (1986). Pulse-chase studies of the synthesis and intracellular transport of apolipoprotein B-100 in Hep G2 cells. J Biol Chem.

[57] Andreev D.E., O'Connor P.B.F., Fahey C., Kenny E.M., Terenin I.M., Dmitriev S.E. (2015). Translation of 5′ leaders is pervasive in genes resistant to eIF2 repression. Elife.

[58] Dana A., Tuller T. (2012). Determinants of translation elongation speed and ribosomal profiling biases in mouse embryonic stem cells. PLoS Comput Biol.

[59] Michel A.M., Andreev D.E., Baranov P.V. (2014). Computational approach for calculating the probability of eukaryotic translation initiation from ribo-seq data that takes into account leaky scanning. BMC Bioinf.

[60] Guttman M., Russell P., Ingolia N.T., Weissman J.S., Lander E.S. (2013). Ribosome profiling provides evidence that large noncoding RNAs do not encode proteins. Cell.

[61] Ingolia N.T., Brar G.A., Stern-Ginossar N., Harris M.S., Talhouarne G.J.S., Jackson S.E. (2014). Ribosome profiling reveals pervasive translation outside of annotated protein-coding genes. Cell Rep.

[62] Cohen S.M. (2014). Everything old is new again: (linc)RNAs make proteins!. EMBO J.

[63] Chew G.-L., Pauli A., Rinn J.L., Regev A., Schier A.F., Valen E. (2013). Ribosome profiling reveals resemblance between long non-coding RNAs and 5′ leaders of coding RNAs. Development.

[64] Li L.-J., Leng R.-X., Fan Y.-G., Pan H.-F., Ye D.-Q. (2017). Translation of noncoding RNAs: focus on lncRNAs, pri-miRNAs, and circRNAs. Exp Cell Res.

[65] Crappé J., Van Criekinge W., Trooskens G., Hayakawa E., Luyten W., Baggerman G. (2013). Combining in silico prediction and ribosome profiling in a genome-wide search for novel putatively coding sORFs. BMC Genomics.

[66] Zupanic A., Meplan C., Grellscheid S.N., Mathers J.C., Kirkwood T.B.L., Hesketh J.E. (2014). Detecting translational regulation by change point analysis of ribosome profiling data sets. RNA.

[67] Artieri C.G., Fraser H.B. (2014). Accounting for biases in riboprofiling data indicates a major role for proline in stalling translation. Genome Res.

[68] Bartholomäus A., Del Campo C., Ignatova Z. (2016). Mapping the non-standardized biases of ribosome profiling. Biol Chem.

[69] Stumpf C.R., Moreno M.V., Olshen A.B., Taylor B.S., Ruggero D. (2013). The translational landscape of the mammalian cell cycle. Mol Cell.

[70] Aramayo R., Polymenis M. (2017). Ribosome profiling the cell cycle: lessons and challenges. Curr Genet.

[71] Brubaker S.W., Gauthier A.E., Mills E.W., Ingolia N.T., Kagan J.C. (2014). A bicistronic MAVS transcript highlights a class of truncated variants in antiviral immunity. Cell.

[72] Cenik C., Cenik E.S., Byeon G.W., Grubert F., Candille S.I., Spacek D. (2015). Integrative analysis of RNA, translation, and protein levels reveals distinct regulatory variation across humans. Genome Res.

[73] Thoreen C.C., Chantranupong L., Keys H.R., Wang T., Gray N.S., Sabatini D.M. (2012). A unifying model for mTORC1-mediated regulation of mRNA translation. Nature.

[74] Hsieh A.C., Liu Y., Edlind M.P., Ingolia N.T., Janes M.R., Sher A. (2012). The translational landscape of mTOR signalling steers cancer initiation and metastasis. Nature.

[75] Bazzini A.A., Johnstone T.G., Christiano R., Mackowiak S.D., Obermayer B., Fleming E.S. (2014). Identification of small ORFs in vertebrates using ribosome footprinting and evolutionary conservation. EMBO J.

[76] Dunn J.G., Foo C.K., Belletier N.G., Gavis E.R., Weissman J.S. (2013). Ribosome profiling reveals pervasive and regulated stop codon readthrough in Drosophila melanogaster. Elife.

[77] Aspden J.L., Eyre-Walker Y.C., Phillips R.J., Amin U., Mumtaz M.A.S., Brocard M. (2014). Extensive translation of small open reading frames revealed by poly-Ribo-Seq. Elife.

[78] Miettinen T.P., Björklund M. (2015). Modified ribosome profiling reveals high abundance of ribosome protected mRNA fragments derived from 3′ untranslated regions. Nucleic Acids Res.

[79] Stadler M., Fire A. (2011). Wobble base-pairing slows in vivo translation elongation in metazoans. RNA.

[80] Juntawong P., Girke T., Bazin J., Bailey-Serres J. (2014). Translational dynamics revealed by genome-wide profiling of ribosome footprints in Arabidopsis. Proc Natl Acad Sci U S A.

[81] Caro F., Ahyong V., Betegon M., DeRisi J.L. (2014). Genome-wide regulatory dynamics of translation in the plasmodium falciparum asexual blood stages. Elife.

[82] Bunnik E.M., Chung D.-W.D., Hamilton M., Ponts N., Saraf A., Prudhomme J. (2013). Polysome profiling reveals translational control of gene expression in the human malaria parasite plasmodium falciparum. Genome Biol.

[83] Jensen B.C., Ramasamy G., Vasconcelos E.J.R., Ingolia N.T., Myler P.J., Parsons M. (2014). Extensive stage-regulation of translation revealed by ribosome profiling of Trypanosoma brucei. BMC Genomics.

[84] Vasquez J.-J., Hon C.-C., Vanselow J.T., Schlosser A., Siegel T.N. (2014). Comparative ribosome profiling reveals extensive translational complexity in different Trypanosoma brucei life cycle stages. Nucleic Acids Res.

[85] Clayton C.E. (2002). Life without transcriptional control? From fly to man and back again. EMBO J.

[86] Stern-Ginossar N., Ingolia N.T. (2015). Ribosome profiling as a tool to decipher viral complexity. Annu Rev Virol.

[87] Khong A., Bonderoff J.M., Spriggs R.V., Tammpere E., Kerr C.H., Jackson T.J. (2016). Temporal regulation of distinct internal ribosome entry sites of the dicistroviridae cricket paralysis virus. Virus.

[88] Bercovich-Kinori A., Tai J., Gelbart I.A., Shitrit A., Ben-Moshe S., Drori Y. (2016). A systematic view on influenza induced host shutoff. Elife.

[89] Gao X., Wan J., Liu B., Ma M., Shen B., Qian S.-B. (2015). Quantitative profiling of initiating ribosomes in vivo. Nat Methods.

[90] Nakahigashi K., Takai Y., Kimura M., Abe N., Nakayashiki T., Shiwa Y. (2016). Comprehensive identification of translation start sites by tetracycline-inhibited ribosome profiling. DNA Res.

[91] Rooijers K., Loayza-Puch F., Nijtmans L.G., Agami R. (2013). Ribosome profiling reveals features of normal and disease-associated mitochondrial translation. Nat Commun.

[92] Zoschke R., Watkins K.P., Barkan A. (2013). A rapid ribosome profiling method elucidates chloroplast ribosome behavior in vivo. Plant Cell.

[93] Chotewutmontri P., Barkan A. (2016). Dynamics of chloroplast translation during chloroplast differentiation in maize. PLoS Genet.

[94] Couvillion M.T., Churchman L.S. (2017). Mitochondrial ribosome (Mitoribosome) profiling for monitoring mitochondrial translation in vivo. Curr Protoc Mol Biol.

[95] Williams C.C., Jan C.H., Weissman J.S. (2014). Targeting and plasticity of mitochondrial proteins revealed by proximity-specific ribosome profiling. Science.

[96] Sotelo-Silveira J.R., Holt C.E. (2014). Introduction to the special issue on local protein synthesis in axons. Dev Neurobiol.

[97] Sotelo-Silveira J.R., Calliari A., Kun A., Koenig E., Sotelo J.R. (2006). RNA trafficking in axons. Traffic.

[98] Calliari A., Farías J., Puppo A., Canclini L., Mercer J.A., Munroe D. (2014). Myosin Va associates with mRNA in ribonucleoprotein particles present in myelinated peripheral axons and in the central nervous system. Dev Neurobiol.

[99] Shigeoka T., Jung H., Jung J., Turner-Bridger B., Ohk J., Lin J.Q. (2016). Dynamic axonal translation in developing and mature visual circuits. Cell.

[100] Hornstein N., Torres D., Sharma Das S., Tang G., Canoll P., Sims P.A. (2016). Ligation-free ribosome profiling of cell type-specific translation in the brain. Genome Biol.

[101] Chung B.Y., Hardcastle T.J., Jones J.D., Irigoyen N., Firth A.E., Baulcombe D.C. (2015). The use of duplex-specific nuclease in ribosome profiling and a user-friendly software package for Ribo-seq data analysis. RNA.

[102] Popa A., Lebrigand K., Paquet A., Nottet N., Robbe-Sermesant K., Waldmann R. (2016). RiboProfiling: a bioconductor package for standard Ribo-seq pipeline processing. F1000Res.

[103] Michel A.M., Mullan J.P.A., Velayudhan V., O'Connor P.B.F., Donohue C.A., Baranov P.V. (2016). RiboGalaxy: a browser based platform for the alignment, analysis and visualization of ribosome profiling data. RNA Biol.

[104] Dunn J.G., Weissman J.S. (2016). Plastid: nucleotide-resolution analysis of next-generation sequencing and genomics data. BMC Genomics.

[105] Wang H., McManus J., Kingsford C. (2016). Isoform-level ribosome occupancy estimation guided by transcript abundance with Ribomap. Bioinformatics.

[106] Calviello L., Mukherjee N., Wyler E., Zauber H., Hirsekorn A., Selbach M. (2016). Detecting actively translated open reading frames in ribosome profiling data. Nat Methods.

[107] Ji Z., Song R., Huang H., Regev A., Struhl K. (2016). Transcriptome-scale RNase-footprinting of RNA-protein complexes. Nat Biotechnol.

[108] Larsson O., Sonenberg N., Nadon R. (2011). anota: analysis of differential translation in genome-wide studies. Bioinformatics.

[109] Zhong Y., Karaletsos T., Drewe P., Sreedharan V.T., Kuo D., Singh K. (2017). RiboDiff: detecting changes of mRNA translation efficiency from ribosome footprints. Bioinformatics.

[110] Xiao Z., Zou Q., Liu Y., Yang X. (2016). Genome-wide assessment of differential translations with ribosome profiling data. Nat Commun.

[111] Legendre R., Baudin-Baillieu A., Hatin I., Namy O. (2015). RiboTools: a galaxy toolbox for qualitative ribosome profiling analysis. Bioinformatics.

[112] Crappé J., Ndah E., Koch A., Steyaert S., Gawron D., De Keulenaer S. (2015). PROTEOFORMER: deep proteome coverage through ribosome profiling and MS integration. Nucleic Acids Res.

[113] Fields A.P., Rodriguez E.H., Jovanovic M., Stern-Ginossar N., Haas B.J., Mertins P. (2015). A regression-based analysis of ribosome-profiling data reveals a conserved complexity to mammalian translation. Mol Cell.

[114] Tebaldi T., Dassi E., Kostoska G., Viero G., Quattrone A. (2014). tRanslatome: an R/Bioconductor package to portray translational control. Bioinformatics.

[115] Liu W., Xiang L., Zheng T., Jin J., Zhang G. (2018). TranslatomeDB: a comprehensive database and cloud-based analysis platform for translatome sequencing data. Nucleic Acids Res.

[116] H Backman T.W., Girke T. (2016). systemPipeR: NGS workflow and report generation environment. BMC Bioinf.

